# Iran’s Coping Experiences with COVID-19: Strategies and Recommendations

**DOI:** 10.1017/dmp.2020.441

**Published:** 2020-11-18

**Authors:** Mahmoudreza Peyravi, Ahmad Soltani, Mahnaz Ahmadi, Milad Ahmadi Marzaleh

**Affiliations:** 1 Department of Health in Disasters and Emergencies, Health Human Resources Research Center, School of Management and Medical Informatics, Shiraz University of Medical Sciences, Shiraz, Iran; 2 Research Center for Emergency and Disaster Resilience, Red Crescent Society of the Islamic Republic of Iran, Tehran, Iran; 3 Clinical Research Development Center, Imam Reza Hospital, Kermanshah University of Medical Sciences, Kermanshah, Iran; 4 Research Center for Health Management in Mass Gathering, Red Crescent Society of the Islamic Republic of Iran, Tehran, Iran; 5 Student Research Committee, Department of Health in Disasters and Emergencies, Health Human Resources Research Center, School of Management and Medical Informatics, Shiraz University of Medical Sciences, Shiraz, Iran; 6 Health Policy Research Center, Institute of Health, Shiraz University of Medical Sciences, Fars, Iran

**Keywords:** contagious disease, corona, COVID-19, Iran, management

The novel coronavirus disease (COVID-19) was first identified in December 2019 in Wuhan, China.^1^ The virus that causes COVID-19 is highly contagious and has a high risk of infection. The World Health Organization declared a global health emergency as the novel coronavirus outbreak spread well beyond China, where it emerged.^1,2^ According to international reports, by February 10, 2020, 43 112 confirmed COVID-19 cases were reported worldwide and the total number of deaths was reported as at least 1018.^3^ Movements of human populations, human-to-human transmission, and environmental factors can transmit the virus. The most common clinical symptoms of the virus include fever and cough, headache, muscle cramps, and fatigue.^4^ However, about 20% of confirmed cases showed severe symptoms and the mortality rate for this virus was reported by approximately 3%.^5^ The first positive case of the disease was announced in Iran on February 18, 2020, and new cases were identified in many Iranian cities. According to reports by the Iranian Ministry of Health and Medical Education, on February 27, 2020, 20 Iranian provinces have been affected with the new coronavirus, and a total of 141 disease cases, 22 deaths, and 54 cured cases were reported. From the earliest days of the outbreak, the Iranian Government has taken valuable measures. However, the main responsibility for its management was given to the Ministry of Health. The following sections describe some of the strategies, weaknesses, and recommendations to better manage these kinds of epidemics.

## Strategies

Strategies include: (1) isolation; (2) quarantine; (3) examining and monitoring all air travelers arriving in Iran; (4) closing educational centers; (5) educating and training the public through media and cyberspace; (6) restricting travels to countries bordering Iran; (7) distributing health packages to families; (8) increasing public media literacy; (9) holding online and virtual training courses of the Iranian Red Crescent Society; (10) disinfecting public transportation; (11) equipping and distributing personal protective equipment among the hospital and prehospital personnel; (12) launching a surveillance system; (13) establishing monitoring, control, and response to COVID-19; (14) holding daily meetings with other organizations in charge; (15) increasing public awareness of COVID-19; (16) managing mass gatherings; (17) giving accurate information to the public; (18) performing online daily tasks; (19) disinfecting the surfaces and environment continuously; and (20) caring for high-risk individuals.

## Weaknesses

During the outbreak, there have been sporadic and irregular notifications and incidences of mis-notification.

## Recommendations

Recommendations include: (1) holding virtual training courses to reduce and manage stress for the public; (2) managing cyber rumors; (3) establishing an active and syndromic care system; (4) considering unified governance and waterfall management; (5) compiling unified training, instructions, protocols, and guidelines; (6) removing financial intermediation and rent-seeking activities and distributing and supplying the required chain of medical equipment; (7) determining job description for the responsible, collaborative, and sponsored organizations; and (8) strengthening training for crisis officers, health authorities, police, and the public.

## Conclusion

After the COVID-19 outbreak spread throughout Iran, productive public engagement and attention to the Iranian Government’s recommendations have contributed to controlling the virus. Public education and personal hygiene are the fundamental factors used to prevent the transmission of COVID-19. Government measures to prevent and control the disease have been favorable; however, with public participation and interorganizational and intraorganizational cooperation with organizations such as the Army, Ministry of Health, the Ministry of Road and Transportation, Red Crescent Society, and other responsible agencies, the control of this disease is possible. Considering the national interests and prioritizing public health, all necessary measures to prevent more people from contracting the virus through the development of uniform instructions, guidelines, and treatment protocols should be taken.
